# Melatonin in Integrative Oncology: Biological Mechanisms, Therapeutic Evidence and Implementation Strategies

**DOI:** 10.32604/or.2026.077020

**Published:** 2026-04-22

**Authors:** Jarosław Nuszkiewicz, Joanna Wróblewska, Marek Jóźwiak, Marta Pawłowska

**Affiliations:** 1 Department of Medical Biology and Biochemistry, Faculty of Medicine, Ludwik Rydygier Collegium Medicum in Bydgoszcz, Nicolaus Copernicus University in Toruń, 24 Karłowicza Street, Bydgoszcz, Poland; 2 Department of Organizational Innovation Management, Faculty of Management, Bydgoszcz University of Science and Technology, 7 Professor S. Kaliski Avenue, Bydgoszcz, Poland

**Keywords:** Adjuvant therapy, integrative oncology, melatonin, organizational strategies, oxidative stress (OS)

## Abstract

Melatonin, an endogenous indoleamine primarily synthesized in the pineal gland, has emerged as a promising adjunctive agent within integrative oncology due to its pleiotropic biological actions. Beyond its well-known chronobiological functions, melatonin exerts potent redox-regulatory, anti-inflammatory, oncostatic, and immune-modulating effects that are relevant across multiple stages of carcinogenesis and cancer therapy. Oxidative stress (OS), defined as an imbalance between reactive oxygen and nitrogen species (ROS/RNS) generation and antioxidant defenses, plays a central role in DNA damage, protein adduct formation, and lipid peroxidation, ultimately contributing to mutation accumulation, treatment resistance, and tumor progression. Melatonin modulates these OS-related processes through both receptor-dependent and receptor-independent mechanisms, including mitochondrial stabilization, enhancement of antioxidant enzyme activity, inhibition of pro-oxidant pathways, regulation of cell-cycle checkpoints, and promotion of apoptosis in malignant cells while protecting healthy tissues. Preclinical studies demonstrate synergistic interactions between melatonin and chemotherapy, radiotherapy, targeted agents, and immunotherapies, with consistent reductions in treatment toxicity and improvements in tumor control. Emerging clinical evidence supports its potential benefits in quality of life, sleep regulation, fatigue, and selected oncologic outcomes, although heterogeneity in dosing, formulations, and study design remains a key limitation. At the organizational and system levels, successful integration of melatonin into oncology practice requires interdisciplinary collaboration, standardized protocols, clinician awareness, regulatory clarity, and evidence-based implementation strategies. The aim of this narrative review is to synthesize current molecular, experimental, and clinical evidence on melatonin in integrative oncology, with particular emphasis on redox-related mechanisms, therapeutic interactions, and implementation challenges.

## 1 Introduction

Cancer remains one of the leading causes of morbidity and mortality worldwide, and despite substantial advances in precision oncology, chemotherapy, radiotherapy and immunotherapy, many patients continue to experience significant treatment-related toxicity, impaired quality of life and insufficient therapeutic response [1–3]. These challenges have increased interest in adjunctive strategies capable of supporting conventional oncologic care by reducing adverse effects, modulating key biological pathways and enhancing overall treatment effectiveness [4]. Among these pathways, oxidative stress (OS) plays a central role in carcinogenesis, tumor progression and treatment resistance. Targeting redox dysregulation has therefore become an attractive concept within integrative oncology [5–7].

Recent epidemiological data further underscore the magnitude of the global cancer burden. According to the most recent estimates, more than two million new cancer cases and over 600,000 cancer-related deaths are projected to occur in the United States alone in 2025, despite a continued long-term decline in age-adjusted cancer mortality rates [8]. Importantly, these favorable mortality trends coexist with a growing burden of disease in younger and middle-aged populations, particularly among women, as well as with persistent racial and socioeconomic disparities in cancer incidence and outcomes [8]. Together, these observations highlight that improvements in survival do not fully offset the increasing complexity of cancer care and reinforce the need for supportive and integrative approaches that may complement standard oncological therapies.

Melatonin, an endogenously produced indoleamine synthesized primarily by the pineal gland, has gained attention as a potential adjunct in cancer care due to its pleiotropic biological activity [9]. Beyond its role in circadian regulation, melatonin exhibits potent antioxidant and pro-oxidant effects depending on cellular context, supports mitochondrial function, modulates immune responses, influences cell-cycle progression and promotes apoptotic pathways in malignant cells [10]. Preclinical research suggests that melatonin may enhance the efficacy of chemotherapeutic agents, sensitize tumors to radiotherapy, reduce toxicity in normal tissues and interact synergistically with targeted and immune-based therapies [11]. These diverse mechanisms make melatonin uniquely positioned at the intersection of molecular oncology, clinical therapeutics and supportive cancer care [12].

Although multiple studies have explored the molecular effects of melatonin or its potential clinical benefits, the existing literature remains fragmented. Many publications focus either on melatonin’s antioxidant properties, its mitochondrial actions or its utility in symptom management, while others examine its preclinical impact on specific cancer types. Only limited attempts have been made to integrate these mechanistic insights with evolving evidence from clinical trials. Moreover, prior reviews rarely address the organizational and systemic factors that shape the implementation of adjunctive therapies in oncology, including clinician awareness, regulatory considerations, workflow integration, quality standards and patient-centered decision-making. As a result, there is a lack of comprehensive, interdisciplinary syntheses that connect biological mechanisms with translational, clinical and health-system perspectives relevant to real-world oncology practice.

This gap is particularly important because even the most promising biologically active compounds require appropriate organizational readiness, standardized protocols and system-level support to be evaluated, recommended and consistently applied in routine care. Melatonin, widely available as an over-the-counter supplement in many countries, presents unique opportunities and challenges for clinical translation, highlighting the need for an integrated review that goes beyond mechanistic or clinical data alone.

Given these considerations, the aim of this narrative review is to provide a comprehensive, interdisciplinary synthesis of current knowledge on melatonin in integrative oncology. We summarize key aspects of redox biology and oncogenic pathways influenced by melatonin, consolidate preclinical and clinical evidence regarding its therapeutic potential and highlight emerging applications in conjunction with established cancer treatments. In parallel, we explore organizational, regulatory and system-level factors that influence how adjunctive therapies such as melatonin can be responsibly incorporated into oncology practice. This dual biomedical and management-oriented perspective seeks to offer a multidimensional understanding of melatonin’s potential role within contemporary cancer care.

The review is organized into major sections covering OS and oncogenic processes, key molecular mechanisms of melatonin, preclinical and clinical evidence, strategic and system-level implementation considerations, and future research directions.

## 2 Oxidative Stress and Cancer

### 2.1 Definition and Mechanisms

OS is a key biological phenomenon underlying both cancer initiation and progression. It results from an imbalance between the production of reactive oxygen and nitrogen species (ROS/RNS) and cellular antioxidant capacity [13]. Under physiological conditions, low levels of ROS act as signaling molecules, regulating metabolic pathways, the cell cycle, and the immune response. However, excessive ROS production disrupts these regulatory functions, leading to cellular structural damage and increasing the likelihood of mutations and permanent genomic changes [14,15].

High concentrations of ROS contribute to nitrogenous base modifications, DNA strand breaks, protein adduct formation, and lipid membrane destabilization. Such molecular damage promotes genomic instability [16]. Mutation accumulation supports cancer cell survival and proliferation. Mitochondria are the primary source of ROS in the cell. Mitochondrial dysfunction is frequently observed in transformed cells [17]. Current research highlights that impaired activity of complexes I and III of the mitochondrial respiratory chain increases electron leakage and stimulates ROS overproduction, constituting an early event in oncogenic transformation [18,19]. Age, chronic inflammation, and metabolic disorders further exacerbate these processes by increasing mitochondrial stress and generating ROS [20–22].

Other important factors contributing to intracellular ROS accumulation include nicotinamide adenine dinucleotide phosphate (NADPH) oxidases and enzymes involved in xenobiotic metabolism, particularly in tissues exposed to chronic inflammation or environmental toxins [23]. In recent years, elevated expression of NADPH oxidases has been identified as a driver of the development of several aggressive malignancies, from pancreatic cancer to gliomas. Under such conditions, endogenous antioxidant systems become overwhelmed [24]. This leads to reduced activity of essential ROS-neutralizing enzymes and to glutathione (GSH) depletion. The resulting chronic redox imbalance exacerbates oxidative damage to DNA, proteins, and lipids, ultimately promoting mechanisms that initiate and sustain carcinogenesis [5].

### 2.2 Role in Carcinogenesis and Progression

**Figure 1:**
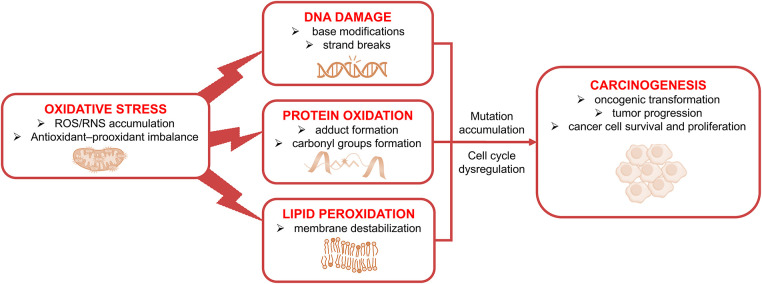
Oxidative stress (OS)-induced macromolecular damage contributing to carcinogenesis. Excessive production of reactive oxygen species (ROS) and reactive nitrogen species (RNS), together with an imbalance between antioxidant and pro-oxidant systems, triggers three major categories of cellular injury: DNA damage (base modifications and strand breaks), protein oxidation (carbonylation, thiol modification, and functional impairment), and lipid peroxidation (membrane destabilization and reactive aldehyde formation). These processes cumulatively promote mutation accumulation, cell-cycle dysregulation, oncogenic transformation, and increased cancer-cell survival and proliferation, thereby establishing a mechanistic foundation for the role of redox imbalance in carcinogenesis and therapy resistance. The figure was created using Microsoft PowerPoint 2021.

OS plays a critical role in all stages of carcinogenesis, influencing tumor initiation, promotion, and progression. At the mechanistic level, redox imbalance contributes to neoplastic transformation through three major categories of macromolecular damage: oxidative DNA damage, protein oxidation with dysregulation of redox-sensitive signaling pathways, and lipid peroxidation–driven alterations in membrane integrity and cellular behavior (see Fig. 1). These interconnected processes promote mutation accumulation, cell-cycle dysregulation, and enhanced cancer-cell survival, thereby establishing a mechanistic foundation for tumor development and progression.

#### 2.2.1 Oxidative Stress-Induced DNA Damage and Genomic Instability

One of the earliest and most critical consequences of excessive ROS production is oxidative DNA damage. Elevated ROS levels induce a broad spectrum of DNA lesions, including the formation of 8-oxo-7,8-dihydro-2^′^-deoxyguanosine (8-oxo-dG), single- and double-strand breaks, and oxidative base modifications, as well as disruption of DNA repair pathways [25]. Persistent oxidative DNA damage promotes genomic instability, a hallmark of cancer, by increasing the frequency of mutations and chromosomal aberrations [26,27].

Mechanistically, oxidized DNA bases such as 8-oxo-dG can mispair during replication, generating mutational hotspots and accelerating mutation accumulation. In parallel, ROS-induced impairment of DNA repair mechanisms and mitochondrial dysfunction interferes with p53-dependent cell-cycle checkpoints, particularly at the G1/S and G2/M transitions, allowing genomically unstable cells to escape apoptosis and continue proliferating [28,29]. Through these mechanisms, OS creates a permissive environment for oncogenic transformation and early clonal expansion during tumor initiation.

#### 2.2.2 Protein Oxidation and Redox-Dependent Dysregulation of Signaling Pathways

Beyond direct genotoxicity, OS promotes carcinogenesis through oxidative modification of proteins and sustained activation of redox-sensitive signaling pathways. Protein oxidation, including thiol oxidation and carbonylation, can alter the activity of kinases, phosphatases, and transcription factors, thereby reshaping intracellular signaling networks that regulate cell survival, proliferation, and inflammation. In cancer cells, such redox-dependent post-translational modifications frequently sustain pro-survival and pro-proliferative signaling cascades [28,30,31].

Chronic OS modulates key oncogenic pathways, including phosphatidylinositol 3-kinase/protein kinase B (PI3K/AKT), hypoxia-inducible factor 1-alpha (HIF-1 α), and nuclear factor kappa-light-chain-enhancer of activated B cells (NF-κB), which collectively regulate proliferation, angiogenesis, metabolic reprogramming, and resistance to apoptosis [28]. During the promotion phase of carcinogenesis, these pathways support the clonal expansion of transformed cells and adaptation to persistently elevated ROS levels [32]. This adaptive phenotype is frequently accompanied by activation of antioxidant defense programs, enabling tumor cells to tolerate continuous OS while simultaneously undergoing additional ROS-induced mutagenesis, further enhancing their aggressive potential [33].

At the mechanistic level, these effects are integrated by redox-sensitive regulatory networks involving nuclear factor erythroid 2–related factor 2 (Nrf2), which governs antioxidant responses and metabolic adaptation, as well as PI3K/AKT- and NF-κB–dependent signaling that promotes survival and chronic inflammation within the tumor microenvironment [30,31]. Together, protein oxidation and redox-driven signaling dysregulation establish a molecular framework that links OS to sustained proliferative signaling and tumor progression.

#### 2.2.3 Lipid Peroxidation, Membrane Remodeling, and Tumor Progression

Lipid peroxidation represents a third major mechanism by which OS contributes to cancer progression and metastatic potential. Oxidative damage to membrane lipids leads to membrane destabilization and the generation of reactive aldehydes, which can further propagate oxidative signaling and modify cellular behavior. These processes alter membrane fluidity, receptor function, and intracellular signaling, thereby facilitating tumor cell adaptation to oxidative environments [34,35].

During later stages of tumor progression, ROS-driven lipid peroxidation and redox signaling promote cancer cell migration, invasiveness, and epithelial–mesenchymal transition (EMT), a key process enabling metastatic dissemination [35]. Molecular analyses indicate that ROS modulate the activity of transcription factors such as Snail, Twist, and zinc finger E-box binding homeobox 1 (ZEB1), thereby facilitating detachment from the primary tumor and colonization of distant tissues [36,37]. Importantly, ROS exert a dual role during metastasis: while moderate ROS levels promote invasion and survival, excessive oxidative stress can induce cytotoxicity and limit tumor cell viability. Thus, OS dynamically shapes tumor progression by simultaneously driving metastatic potential and imposing redox-dependent constraints on tumor growth [34].

### 2.3 Oxidative Stress in Therapy

OS represents a central determinant of both therapeutic efficacy and toxicity in contemporary oncology [38]. A growing body of clinical and translational evidence indicates that redox imbalance critically influences responses to chemotherapy, radiotherapy, and selected targeted therapies, shaping tumor sensitivity, treatment resistance, and the severity of adverse effects [39,40]. Importantly, the role of ROS in cancer therapy is inherently dual, encompassing both acute cytotoxic signaling that mediates tumor cell killing and chronic adaptive responses that promote tumor survival and resistance. Understanding this balance is essential for the rational design of redox-modulating therapeutic strategies.

#### 2.3.1 Acute Therapy-Induced Reactive Oxygen Species: Cytotoxic Damage and Tumor Cell Death

Many conventional anticancer therapies, including cytotoxic chemotherapy and radiotherapy, rely on increased intracellular ROS generation as a primary mechanism of tumor cell killing [41]. Therapy-induced ROS cause extensive macromolecular damage, affecting DNA, proteins, and lipids, and thereby triggering apoptosis, ferroptosis, or necrotic cell death [41]. Due to their intrinsically disrupted redox homeostasis, cancer cells are particularly vulnerable to acute ROS overload, which overwhelms antioxidant defenses and compromises mitochondrial integrity [42].

Radiotherapy represents a classical example of acute ROS-driven cytotoxicity. Ionizing radiation generates high levels of ROS that induce massive DNA damage, including strand breaks and oxidative base lesions, leading to effective tumor cell eradication [43,44]. However, even at this early stage, ROS signaling initiates compensatory stress responses that may influence subsequent therapeutic outcomes [43,44]. These acute cytotoxic effects of ROS correspond to the macromolecular damage pathways illustrated in Fig. 1 and form the mechanistic basis of redox-dependent tumor cell killing.

#### 2.3.2 Chronic Reactive Oxygen Species Exposure: Redox Adaptation and Therapy Resistance

In contrast to acute ROS bursts, sustained or sublethal ROS exposure promotes adaptive responses that enable tumor cells to survive therapeutic stress. Cancer cells exposed to chronically elevated ROS levels activate endogenous defense mechanisms, including increased glutathione synthesis, upregulation of peroxidase activity, and transcriptional reprogramming mediated by the NRF2 pathway [45].

Under oxidative conditions, disruption of the NRF2–Keap1 interaction leads to NRF2 stabilization and nuclear translocation, where it induces the expression of antioxidant, detoxifying, and cytoprotective genes. This adaptive redox reprogramming enhances ROS neutralization, supports metabolic flexibility, and contributes to resistance against ROS-inducing therapies [45]. Such mechanisms have been documented in both experimental models and patient tumor samples and are increasingly recognized as clinically relevant drivers of reduced therapeutic efficacy.

Chronic OS further reinforces tumor survival by stimulating DNA repair pathways and modulating cellular programs associated with EMT, thereby facilitating invasion, metastasis, and long-term disease progression [46,47]. Thus, while acute ROS signaling promotes tumor cell death, persistent redox stress fosters selective pressure that favors resistant and more aggressive tumor cell populations.

#### 2.3.3 Context Dependency: Cancer Type—Specific Redox States and Therapeutic Response

The balance between ROS-mediated cytotoxicity and adaptive resistance is highly context dependent and varies markedly across cancer types. Tumors differ in baseline ROS levels, mitochondrial function, antioxidant capacity, and reliance on redox-sensitive signaling pathways, resulting in heterogeneous responses to ROS-inducing therapies. In some malignancies, high intrinsic oxidative stress predisposes cells to ROS-driven apoptosis, whereas in others, robust antioxidant systems confer pronounced resistance to treatment [48].

These cancer type–specific redox states critically influence therapeutic outcomes and contribute to intertumoral variability in sensitivity, toxicity, and resistance patterns. Consequently, redox-modulating strategies must be tailored to the biological context of each tumor entity to maximize therapeutic benefit while minimizing collateral damage to healthy tissues [49,50].

Detailed molecular mechanisms underlying redox modulation by melatonin, including mitochondrial signaling and cancer-type–specific responses, are discussed in Section 3.

Table 1 categorizes key ROS-related therapeutic mechanisms according to cancer type, treatment modality, dominant redox pathways involved, and their functional consequences, providing a concise overview that complements the mechanistic framework outlined above.

**Table 1:** Dual roles of OS in cancer therapy: acute cytotoxicity vs. chronic adaptive responses.

Therapy context/cancer type	Dominant ROS pattern	Key molecular pathways involved	Biological consequence	Clinical implication	Ref.
Chemotherapy, radiotherapy (solid tumors)	Acute ROS overload	Mitochondrial dysfunction; DNA damage response	Apoptosis, ferroptosis, tumor cell death	Increased therapeutic efficacy	[41,43,44]
Radiotherapy (radio-sensitive tumors)	Acute ROS generation followed by stress signaling	DNA double-strand breaks; redox-sensitive signaling pathways	Efficient tumor cell killing with parallel activation of stress responses	Initial response followed by potential resistance	[43,44]
Chemotherapy-exposed tumors (various types)	Chronic ROS elevation	NRF2/Keap1 axis; GSH synthesis; antioxidant enzymes	Adaptive redox homeostasis; survival under oxidative stress	Development of treatment resistance	[45–47]
Advanced and metastatic cancers	Dynamic ROS fluctuations	EMT-associated pathways; DNA repair activation; inflammatory signaling	Increased invasiveness and metastatic potential	Disease progression despite therapy	[46,47]
Normal tissues during anticancer therapy	Therapy-induced ROS	Lipid peroxidation; mitochondrial damage	Cardiotoxicity, neuropathy, mucositis	Dose-limiting toxicity	[49,50]

Note: Abbreviations used: EMT—epithelial–mesenchymal transition; GSH—glutathione; Keap1—Kelch-like ECH-associated protein 1; NRF2—nuclear factor erythroid 2–related factor 2; OS—oxidative stress; ROS—reactive oxygen species.

## 3 Melatonin: Mechanisms, Pharmacology, and Safety

### 3.1 Molecular Structure and General Mechanisms of Melatonin Action

Melatonin (N-acetyl-5-methoxytryptamine), the main secretory product of the pineal gland synthesized from serotonin (5-hydroxytryptamine), is a molecule widely distributed in nature and performing many diverse functions. Its amphiphilic character and specific properties arise from the presence of two key functional groups, the 5-methoxy group and the N-acyl group, which confer high stability and the ability to cross morphophysiological barriers [51,52]. Melatonin freely diffuses, reaching all cells of the organism, where it influences various physiological processes [53]. The 5-methoxy and N-acyl groups are also responsible for its antioxidant activity. The electron-donating indole ring promotes the scavenging of free radicals, and the presence of these groups enhances this function, providing the molecule with stability and amphiphilicity [51,54]. Melatonin acts as an effective scavenger of ROS and RNS, and also stimulates the expression of antioxidant enzymes: superoxide dismutase (SOD), catalase (CAT), and glutathione peroxidase (GPx). This contributes to maintaining redox balance and protecting DNA from oxidative damage [55]. Studies on melatonin analogs show that the presence of the 5-methoxy group enhances its cytoprotective properties under *in vitro* conditions [54]. Both the 5-methoxy group and the N-acyl chain are required for strong binding to melatonin receptors. The removal of either group decreases affinity and reduces the effectiveness of receptor activation [56].

**Figure 2:**
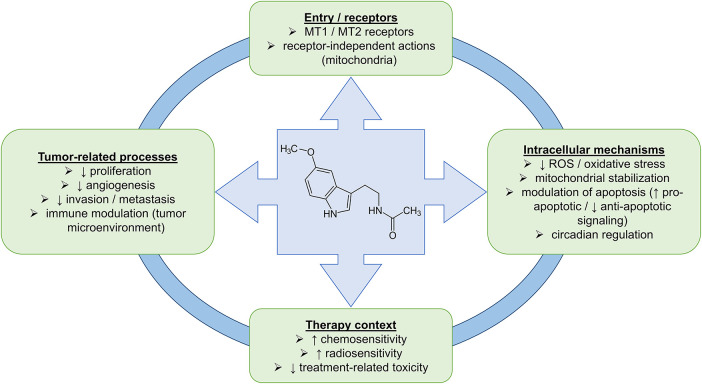
Schematic overview of melatonin structure and its proposed mechanisms of action in cancer. The figure illustrates receptor-dependent (MT1/MT2-mediated) and receptor-independent actions of melatonin, including its effects on mitochondrial function, OS, apoptosis, and circadian regulation. These intracellular mechanisms are linked to key tumor-related processes, such as proliferation, angiogenesis, invasion/metastasis, and immune modulation, as well as to the therapeutic context, including chemosensitivity, radiosensitivity, and treatment-related toxicity. The figure was created using Microsoft PowerPoint 2021.

Melatonin primarily acts through the membrane receptors MT1 and MT2, which are coupled to G proteins. Their activation triggers various signaling pathways depending on the cell type and physiological context. The best-characterized mechanism involves coupling to Gi/o proteins and inhibition of adenylyl cyclase, leading to decreased levels of cAMP and reduced protein kinase A (PKA) activity [57,58]. Activation of MT1 and MT2 receptors modulates intracellular signaling, including pathways such as the mitogen-activated protein kinase/extracellular signal-regulated kinase (MAPK/ERK) cascade, leading to changes in cell proliferation, differentiation, and survival. Melatonin increases the expression of p53 and p21, promoting apoptosis initiation in cancer cells [55]. In addition to MT1 and MT2 receptors, melatonin also interacts with other receptor proteins, thereby expanding the spectrum of its actions. The MT3 receptor, identified as the enzyme quinone reductase 2 (QR2), is present in tissues such as muscle, brown adipose tissue, liver, kidney, heart, lungs, and intestine. QR2 contributes to antioxidant protection by inhibiting electron transfer in quinone-related reactions, thus reducing OS [59]. Melatonin also interacts with the nuclear retinoid-related orphan receptor alpha (RORα), also known as the retinoid Z receptor alpha (RZRα), which regulates the expression of genes involved in circadian rhythm, immune responses, and cell proliferation [59]. A schematic overview summarizing the major molecular and cellular mechanisms through which melatonin may influence tumor biology and therapeutic responses is presented in Fig. 2.

### 3.2 Modulation of Signaling Pathways by Melatonin in Cancer

The wide range of melatonin’s signaling interactions underlies its diverse biological effects. In the context of cancer, these include processes such as cell proliferation, survival, metabolism, interactions with the tumor microenvironment, migration, metastasis, and the protection of healthy tissues. Detailed data on individual signaling pathways, their biological consequences, and experimental models are summarized in Table 2.

**Table 2:** Modulation of signaling pathways by melatonin in cancer models.

Signaling pathway	Biological consequences	Experimental model	Ref.
PI3K/AKT/mTOR	↑ apoptosis (↑ Bax, ↓ Bcl-2, ↑ cytochrome c release, ↑ cleaved caspase activation), ↓ proliferation, ↓ migration, ↓ invasion	GBC cell lines, mouse xenograft model	[60]
PI3K/AKT/mTOR	↑ apoptosis (enhanced by ER stress; associated with ↓ Bcl-2, ↑ Bax, ↑ p-PERK, ↑ p-eIF2 α)	Melanoma cell line B16F10	[61]
AKT–MDM2	Cell cycle arrest at G1/S; ↑ mitochondrial apoptosis (↓ Bcl-xL, ↑ Bax, ↑ cleaved caspase-9, ↑ cleaved caspase-3)	Human GC cell line (SGC-7901)	[63]
ZNF746–PI3K/AKT–MMP-9 axis	↑ apoptosis; ↓ ROS and MMPs; changes in their expression elicit immunomodulation; ↓ migration, cell growth, and invasion	Human bladder cancer cell lines (HT1197, HT1376, T24, RT4); mouse xenograft model	[64]
SIRT3/PDH	↑ activity of mitochondrial respiratory chain complexes I and IV → ↑ ATP production → ↑ mitochondrial ROS generation → induction of apoptosis and inhibition of tumor growth (reversal of the Warburg effect)	LC cell lines (A549, PC9, LLC)	[65]
MT1–HIF-1α/VEGF/VEGFR2 and TGF-β1	↑ MT1 expression → ↓ HIF-1 α; ↓ VEGF and VEGFR2; ↓ TGF-β1 → ↓ angiogenesis (↓ tumor growth)	Serous papillary ovarian carcinoma in ethanol-preferring rats (DMBA-induced)	[72]
NLRP3 inflammasome	↓ angiogenesis and lymphangiogenesis; ↓ tumor growth	A549 lung adenocarcinoma cells maintained in mono-culture or co-cultured with THP-1 macrophages	[67]
FGF19/FGFR4	↑ apoptosis (↑ Bax/Bcl-2 ratio; ↑ cleaved caspase-9; ↑ cleaved caspase-12); ↓ proliferation (↓ tumor growth)	HNSCC cell lines (HN6, HN12, HN30)	[68]

Note: Abbreviations used: AKT—protein kinase B; ATP—adenosine triphosphate; Bax—Bcl-2-associated X protein; Bcl-2—B-cell lymphoma 2; Bcl-xL—B-cell lymphoma-extra large; DMBA—7,12-dimethylbenz[a]anthracene; eIF2α—eukaryotic translation initiation factor 2 α; ER—endoplasmic reticulum; FGF19—fibroblast growth factor 19; FGFR4—fibroblast growth factor receptor 4; GBC—gallbladder cancer; GC—gastric cancer; HIF-1α—hypoxia-inducible factor 1-alpha; HNSCC—head and neck squamous cell carcinoma; LC—lung cancer; MDM2—murine double minute 2; MMP—matrix metalloproteinase; mTOR—mammalian target of rapamycin; MT1—melatonin receptor type 1; NLRP3—NOD-like receptor family pyrin domain-containing 3; PDH—pyruvate dehydrogenase; PERK—PKR-like endoplasmic reticulum kinase; PI3K—phosphoinositide 3-kinase; SIRT3—sirtuin 3; TGF-β1—transforming growth factor beta 1; THP-1—human monocytic cell line; VEGF—vascular endothelial growth factor; VEGFR2—vascular endothelial growth factor receptor 2; ZNF746—zinc finger protein 746.

In the regulation of cancer cell proliferation and survival, a key role is played by the phosphoinositide 3-kinase/protein kinase B/mammalian target of rapamycin (PI3K/AKT/mTOR) signaling pathway, whose activation promotes cell growth, migration, and invasiveness [60–62]. This pathway, which includes the p110 catalytic and p85 regulatory subunits, is frequently overactivated in various cancers, including melanoma, and represents an important therapeutic target [61]. This mechanism involves the regulatory link between AKT, murine double minute 2 (MDM2), and p53, which controls the stability of the p53 protein and influences the cell’s decision between apoptosis and continued cell cycle progression [63]. In bladder cancer, the phosphoinositide 3-kinase/protein kinase B–matrix metalloproteinase-9 (PI3K/AKT–MMP-9) axis plays a crucial role by regulating the expression of matrix metalloproteinases (MMPs), which degrade components of the extracellular matrix and facilitate tumor invasion [64]. Another key area of melatonin action is cellular metabolism. By activating the sirtuin 3/pyruvate dehydrogenase (SIRT3/PDH) axis, melatonin reverses the Warburg effect, shifting cancer cell metabolism from aerobic glycolysis to oxidative phosphorylation and reducing their energetic advantage [65]. The tumor microenvironment also plays an important role, as melatonin influences angiogenesis by inhibiting the vascular endothelial growth factor/vascular endothelial growth factor receptor (VEGF/VEGFR) pathway and, under OS conditions, the HIF-1 α, thereby limiting the formation of new blood vessels within the tumor [66], and modulates inflammatory signaling by blocking the NOD-like receptor family pyrin domain containing 3 (NLRP3) inflammasome, which reduces the secretion of interleukin-1 beta (IL-1β) and interleukin-18 (IL-18), key pro-inflammatory cytokines associated with the tumor microenvironment [67]. EMT also plays an important role in tumor progression. In this process, the activity of the fibroblast growth factor 19/fibroblast growth factor receptor 4 (FGF19/FGFR4) axis promotes cell migration and invasion, while melatonin, by modulating this pathway, may influence the metastatic potential of cancer cells [68]. Activation of MT1/MT2 receptors also affects ionic signaling. Through the Gi/o pathway, it opens G-protein-coupled inwardly rectifying potassium (GIRK) channels, leading to membrane hyperpolarization and a decrease in cellular excitability [69]. It has also been shown that melatonin downregulates transient receptor potential canonical 6 (TRPC6) calcium channels in triple-negative breast cancer cells, thereby impairing calcium entry and reducing cell proliferation and migration [70].

Its action also includes the protection of the hematopoietic system from the toxicity of anticancer therapy. Activation of the PI3K/AKT signaling pathway leads to the inhibition of the caspase cascade, thereby protecting megakaryocytes from doxorubicin-induced apoptosis and supporting the regeneration of hematopoiesis following radio- and chemotherapy [71].

### 3.3 Mitochondrial Function and Oxidative Stress Modulation in Melatonin’s Anticancer Activity

#### 3.3.1 Mitochondrial Apoptosis and Therapy Sensitization

The anticancer activity of melatonin is often associated with its ability to block key signaling pathways [60,61,67,70] and inhibit axes that promote cell proliferation and survival [63,73]. In addition to the classical mechanisms associated with MT1, MT2, and MT3 receptors, as well as nuclear RZR/RORα receptors, melatonin significantly influences the regulation of mitochondrial function [59,74].

In studies on gallbladder cancer cells and on melanoma cells exposed to endoplasmic reticulum stress, melatonin treatment was associated with increased expression of Bcl-2–associated X protein (Bax) and decreased expression of B-cell lymphoma 2 (Bcl-2). In gallbladder cancer, this modulation led to mitochondrial membrane destabilization, cytochrome c release, and caspase activation, indicating the involvement of the mitochondrial apoptotic pathway [60,61]. Although several studies have reported anti-apoptotic or cytoprotective effects of melatonin, these findings refer to non-cancerous tissues exposed to physiological or treatment-related stress. Radogna et al. [75] showed that melatonin reduces susceptibility to apoptosis in immune cells by modulating Bax/Bcl-2 dynamics through 5-hydroxyeicosatetraenoic acid (5-HETE), a metabolite generated via the 5-lipoxygenase pathway. Likewise, in radiation-induced oral mucositis, melatonin protects healthy epithelial cells by limiting mitochondrial damage, inhibiting NLRP3 inflammasome activation, and reducing excessive apoptosis [76]. Importantly, these cytoprotective actions do not contradict melatonin’s well-documented pro-apoptotic effects in malignant cells, where it promotes mitochondrial dysfunction, increases ROS levels, activates caspases, and sensitizes tumor cells to therapy.

#### 3.3.2 Redox Modulation and Oxidative Stress as an Anticancer Mechanism

Studies have confirmed that the antitumor effect of melatonin is associated with the modulation of OS. The increase in ROS production is responsible for inducing apoptosis, while their neutralization abolishes this effect [60,62]. In melanoma cells exposed to endoplasmic reticulum stress, melatonin reduced the expression of antioxidant enzymes such as CAT, Cu/Zn-SOD, and Mn-SOD, thereby favoring the maintenance of OS and enhancing apoptosis [61]. In *in vivo* models, melatonin exhibits strong redox-modulating activity. In chemically induced carcinogenesis in the liver and breast, melatonin reduced thiobarbituric acid reactive substances (TBARS), normalized the activity of antioxidant enzymes (SOD, CAT, GPx, GST), and increased the levels of GSH and vitamins C and E. This led to the restoration of redox homeostasis, reduction of OS, and inhibition of tumor development [77,78]. Importantly, in the breast cancer model, prophylactic administration of melatonin completely prevented tumor formation, confirming its chemopreventive potential [78]. In the bladder cancer model, it was further demonstrated that melatonin reduces mitochondrial membrane potential, indicating activation of the mitochondrial pathway of apoptosis [64]. In different cancer types, melatonin modulates mitochondrial metabolism, leading to its reprogramming and increased susceptibility to apoptosis.

#### 3.3.3 Mitochondrial Metabolism and Bioenergetic Reprogramming

In lung cancer, activation of the SIRT3/PDH axis enhances mitochondrial bioenergetics and shifts metabolism toward oxidative phosphorylation. A similar effect was observed in ovarian cancer, where melatonin reduced mitochondrial membrane potential and reversed the Warburg effect, thereby limiting proliferation and promoting cell death [65,79].

#### 3.3.4 Intramitochondrial Melatonin: Synthesis, Transport, and Local Accumulation

Mitochondria are involved not only in melatonin metabolism but can also synthesize it locally, depending on the cellular demand. Its concentration in mitochondria exceeds that in the blood, which is associated with the need for protection against OS generated by the respiratory chain [80]. The enzyme arylalkylamine N-acetyltransferase (AANAT), which converts serotonin into N-acetylserotonin, is localized in mitochondria, where the presence of acetyl-CoA promotes efficient hormone synthesis [81]. In addition to local synthesis, melatonin is actively transported into mitochondria by the peptide transporters 1 and 2 (PEPT1 and PEPT2), enabling its accumulation at concentrations much higher than in plasma [81]. Huo et al. [82] demonstrated that the presence of PEPT1/2 in the mitochondrial membrane of cancer cells facilitates melatonin accumulation, which leads to mitochondrial membrane potential loss, cytochrome c release, an increased Bax/Bcl-2 ratio, caspase-3 activation, and enhanced apoptosis. This metabolite induces mitochondrial membrane depolarization, increases ROS levels, and activates caspases, contributing to the proapoptotic response in cancer cells, while showing no apoptotic effect in normal cells [83]. Cucielo et al. [79] reported that local synthesis and accumulation of melatonin within mitochondria contribute to the regulation of mitochondrial integrity and cellular energy metabolism in ovarian cancer cells. In this way, the hormone modulates mitochondrial function and alters the metabolic phenotype of cancer cells. Importantly, melatonin promoted cytotoxicity and apoptosis, effects that were even more pronounced after MT1 receptor silencing. The biological effects of melatonin in cancer models include the inhibition of cell proliferation and the reduction of tumor growth *in vivo* without significant adverse effects. Moreover, melatonin can enhance ROS generation in cancer cells, which contributes to the induction of apoptosis, highlighting the context-dependent nature of its action [60,65].

#### 3.3.5 Immunomodulation and Anti-Inflammatory Effects Linked to Mitochondrial Redox Balance

Melatonin also plays an important role in the regulation of the immune response, exhibiting anti-inflammatory and immunomodulatory effects. It has been shown to inhibit the activation of the transcription factor NF-κB and the NLRP3 inflammasome, leading to reduced activation of caspase-1 and decreased secretion of pro-inflammatory cytokines such as IL-1β and IL-18. By stabilizing mitochondrial function and limiting the production of ROS, melatonin suppresses excessive inflammatory responses and reduces tissue damage [51,67,76]. In addition, it modulates the expression of pro-inflammatory enzymes such as cyclooxygenase-2 (COX-2) and inducible nitric oxide synthase (iNOS), resulting in the reduction of inflammatory mediators [51]. Importantly, Lai et al. [84] demonstrated that melatonin increases the expression of sirtuin 1 (SIRT1) in glioblastoma multiforme cells and, through SIRT1 activation, attenuates IL-1β-induced expression of the chemokine C-C motif ligand 2 (CCL2, also known as MCP-1) and the adhesion molecules vascular cell adhesion molecule-1 (VCAM-1) and intercellular adhesion molecule-1 (ICAM-1). This leads to reduced monocyte adhesion to glioblastoma multiforme cells and may help prevent the establishment of an immunosuppressive tumor microenvironment. These findings indicate that melatonin modulates tumor–immune interactions in glioblastoma multiforme cells via SIRT1-dependent pathways. Moreover, melatonin has been reported to influence metabolic activity and OS responses in glioblastoma cells, effects that could contribute to its overall anticancer potential. Lee et al. [85] demonstrated that melatonin inhibits the activity of the mechanistic target of rapamycin complex 1 (mTORC1) and subsequently reduces c-Myc protein levels in hepatocellular carcinoma cells. This leads to the downregulation of key glycolytic enzymes such as hexokinase 2 (HK2) and lactate dehydrogenase A (LDHA), resulting in suppressed glycolysis and reduced proliferation of cancer cells. In turn, Laothong et al. [86] showed that in human cholangiocarcinoma cell lines, treatment with high pharmacological concentrations of melatonin (0.5, 1 and 2 mM) increased ROS production, oxidative DNA damage, and mitochondrial apoptosis, suggesting a potential pro-oxidant effect under these *in-vitro* conditions.

### 3.4 Melatonin as a Component of Chronotherapy and Combination Therapy in Oncology

#### 3.4.1 Pharmacokinetics, Formulations, and Safety of Melatonin in Oncology

Melatonin has long been recognized primarily as a regulator of the circadian rhythm; however, its potential anticancer properties are increasingly being explored. In oncology, melatonin has been investigated as an adjuvant therapy in various dosing regimens. The formulation and release profile of melatonin may influence its pharmacokinetics and therapeutic effects. Immediate-release formulations allow for a rapid increase in circulating melatonin levels and are typically preferred when a prompt adjustment of the circadian rhythm is required, such as in patients with sleep–wake disturbances [80]. The amphiphilic nature of melatonin facilitates its passage through cell membranes and biological barriers, including the blood–brain barrier, which allows it to act both centrally and peripherally. This property enables melatonin to reach various tissues and exert diverse physiological effects through both receptor-dependent and receptor-independent mechanisms [53]. In recent years, intensive research has also been conducted to develop new melatonin receptor ligands with greater selectivity toward MT1 and MT2 receptors and an improved pharmacokinetic profile. The aim of these studies is to create compounds with a prolonged duration of action and higher metabolic stability, which could enhance the effectiveness of therapies based on modulation of the melatonergic system [87]. Melatonin is characterized by rapid absorption and a short time to reach maximum plasma concentration (T_max_ below 30 min), which results from its short half-life and extensive hepatic metabolism involving cytochrome P450 enzymes, mainly CYP1A1, CYP1A2, and CYP2C19. These metabolic processes produce 6-hydroxymelatonin, which is subsequently conjugated and excreted by the kidneys in the form of 6-glucuronylmelatonin and 6-hydroxymelatonin. This process may be impaired in patients with liver diseases, leading to reduced melatonin clearance [55,88]. Melatonin exhibits a very favorable safety profile, with the incidence of adverse effects comparable to that of placebo. The most common symptoms are mild and transient, such as daytime sleepiness, headache, and dizziness. The risk of serious adverse reactions or dependence is minimal. Because melatonin is metabolized by cytochrome P450 enzymes (CYP1A1, CYP1A2, CYP2C19), interactions with drugs such as fluvoxamine, warfarin, and nifedipine are possible. In patients with liver disease, reduced clearance can lead to elevated serum concentrations. In older adults, particularly those using multiple medications, caution and monitoring for potential interactions are advised. Despite these considerations, melatonin is widely regarded as a well-tolerated compound with a low risk of adverse effects, although further studies are needed to assess its long-term safety [88].

#### 3.4.2 Circadian Rhythm Regulation and Chronotherapy-Based Strategies

Prolonged-release melatonin formulations more closely mimic the physiological kinetics of the hormone, providing a gradual release over 4 to 5 h, which may support better regulation of the circadian rhythm and improve sleep quality [80]. In the randomized, double-blind clinical trial MIRCIT involving patients with advanced non-small cell lung cancer (NSCLC), melatonin administered at doses of 10–20 mg per day did not significantly affect overall survival or the incidence of chemotherapy-related adverse events, while a trend toward improved quality of life was observed. In addition, stabilization of OS markers (like 8-oxo-dG) was noted in the melatonin-treated groups, whereas in the placebo group their levels increased and were associated with poorer prognosis. These findings suggest a protective effect of melatonin on healthy cells during cytotoxic therapy and support its safety profile as a potential adjuvant treatment [89]. An increasing body of evidence indicates that disruption of the circadian rhythm is an important factor promoting tumor progression. The regulation of clock gene expression, including *PER1*, *PER2*, *BMAL1*, and *NPAS2*, plays a crucial role in maintaining cellular homeostasis by controlling the cell cycle, DNA repair, and apoptosis. Circadian rhythm disturbances observed in cancer patients, including those with lung cancer, lead to deregulation of these genes, resulting in loss of cell proliferation control, increased genomic instability, and reduced efficiency of repair mechanisms [90,91]. Genes such as *PER2*, *BMAL1*, and *CRY2* act as tumor suppressors, and their reduced expression promotes cancer cell proliferation and the development of treatment resistance, whereas their activation has been associated with the inhibition of tumorigenesis and may enhance the sensitivity of cancer cells to chemotherapy [92].

#### 3.4.3 Melatonin in Combination Therapy: Molecular Mechanisms and Synergistic Effects

Melatonin sensitizes human glioma cells to TRAIL-induced apoptosis by inhibiting the protein kinase C/protein kinase B (PKC/AKT) signaling pathway, which increases death receptor 5 (DR5) levels and decreases the antiapoptotic proteins survivin and Bcl-2 [93]. Studies have developed a modified form of melatonin targeted to mitochondria, in which the melatonin molecule is conjugated with a triphenylphosphonium (TPP) cation, allowing its selective accumulation within the mitochondria of cancer cells. This targeting enhances the anticancer efficacy of melatonin by increasing OS, disrupting mitochondrial function, and inducing pyroptosis in castration-resistant prostate cancer cells [94]. The development of synthetic melatonin receptor agonists such as agomelatine, ramelteon, and tasimelteon demonstrates the therapeutic potential of targeting melatoninergic pathways, particularly in the regulation of circadian rhythms and the treatment of neuropsychiatric disorders. Moreover, preclinical studies suggest that modulation of melatonin receptors may influence OS, inflammatory responses, and apoptotic pathways, indicating a possible role for melatonin-based strategies as supportive approaches in oncology [87]. Melatonin may also act as an effective agent enhancing the activity of anticancer drugs by modulating various signaling pathways responsible for tumor cell growth, survival, and resistance. It has been shown that, in combination with everolimus (an inhibitor of the PI3K/AKT/mTOR pathway) or sorafenib (a tyrosine kinase inhibitor), this compound enhances apoptosis and inhibits tumor cell proliferation through the modulation of the PI3K/AKT/mTOR and platelet-derived growth factor receptor beta/signal transducer and activator of transcription 3 (PDGFR-β/STAT3) pathways [95,96]. Similar effects have also been observed in hematological malignancies, where melatonin, in combination with cytarabine or ABT-737, increased the sensitivity of leukemia cells to treatment by inducing endoplasmic reticulum stress, enhancing OS, and activating autophagy—processes associated with increased apoptosis [97]. In Table 3, a summary of the mechanisms of action of melatonin in combination with selected anticancer drugs is presented. Moreover, in ovarian cancer cells, it has been demonstrated that melatonin not only induces OS and apoptosis but also enhances the effectiveness of cisplatin. In the OVCAR-3 cell line, its addition led to a reduction in the IC50 (half maximal inhibitory concentration) value of this drug, increased caspase-3 activation, and enhanced apoptosis, confirming the synergistic effect of both compounds through the inhibition of the PI3K/AKT pathway [62]. Studies on lung cancer models indicate that melatonin can enhance the antitumor activity of other bioactive compounds such as berberine, thereby supporting its potential role as an adjuvant agent in combination therapy [73].

**Table 3:** Impact of melatonin combined with anticancer drugs on cellular processes.

Type of Cancer/ Experimental Model	Chemothe Rapeutic Agent	Melatonin Dosage	Molecular Mechanisms and Signaling Pathways	Effect on Cellular Processes	Ref.
Breast cancer MCF-7 cells	Everolimus (30 nM)	3 mM	PI3K/AKT/mTOR → inhibition of phosphorylation of p70S6K and 4E-BP1	Increased proportion of Sub-G1 phase cells, suggesting DNA fragmentation consistent with apoptosis; Activation of the autophagy process (increased LC3-II levels and decreased p62 expression); Melatonin combined with everolimus improves mitochondrial function by restoring cellular respiration inhibited by everolimus	[95]
Pancreatic cancer MIAPaCa-2 and PANC-1 cells	Sorafenib (1–10 μM)	0.1, 1, and 2 mM	PDGFR-β/STAT3 → inhibition of PDGFR-β and STAT3 phosphorylation; melatonin may act, at least in part, through MT1/MT2 receptors	Increased apoptotic cell population, caspase-3 activation, cytochrome c release, and loss of mitochondrial membrane potential; Suppression of tumor cell proliferation; Activation of mitochondrial apoptosis in cancer cells without significant systemic toxicity	[96]
Ovarian cancer OVCAR-3 cells	Cisplatin (8.3, 10.4, and 12.6 µM)	4 mM	PI3K/AKT → inhibition of AKT phosphorylation; downregulation of HIF-1 α, VEGF, and p-GSK3β; upregulation of p53	Induction of apoptosis via caspase-3 activation and p53 upregulation; Reduction of cell viability and proliferation; OS with increased ROS generation induced by combined treatment; Downregulation of angiogenesis-related factors (HIF-1 α and VEGF)	[62]
Glioblastoma U87MG cells	Temozolomide (0.8 mM)	1 mM and 3 mM	Regulation of mitochondrial transcription factors (TFAM, TFB1M, TFB2M)	Increased proportion of apoptotic cells through ROS-dependent mitochondrial depolarization; Downregulation of mitochondrial transcription factors (TFAM, TFB1M, TFB2M)	[98]
Acute myeloid leukemia MV4-11 cells	Cytarabine (0.4 and 1 µM); ABT-737 (20 and 80 nM)	1 mM	ER stress → PERK–eIF2α–ATF4–CHOP; Modulation of ER stress markers (BiP, PDI); Activation of autophagy (LC3A/B); ↑ ROS generation; Mitochondrial membrane depolarization; ↑ cytosolic Ca^2+^	Reduction of cell proliferation/viability; Increased apoptotic/dead cell population; Enhanced sensitivity to cytarabine and ABT-737	[97]
Lung cancer Experimental cell models	Berberine (20, 50, 100, and 200 µM)	1 mM	Inhibition of AP-2β/hTERT, NF-κB/COX-2, and Akt/ERK; Activation of caspase/Cyto C signaling; ↑ ROS generation; mitochondrial dysfunction	Increased apoptotic cell population; Reduction of tumor cell proliferation; Enhanced antitumor activity of berberine	[73]
Colorectal cancer Caco-2 cells	Doxorubicin (1 μM)	2 mM	Downregulation of BCL-2 and SURVIVIN; upregulation of BAX and SMAC; inhibition of MMP-2 and MMP-9; Induction of apoptosis via increased BAX and SMAC expression and decreased BCL-2 and SURVIVIN levels	Reduction of cell viability and proliferation in concentration and time dependent manner; Decreased tumor spheroid formation and size; Suppression of cancer cell migration and invasion through downregulation of MMP-2 and MMP-9	[99]
Colorectal cancer SNU-C5/Oxal-R cells (oxaliplatin-resistant)	Oxaliplatin (1 μM)	0.5 mM	Inhibition of PrPC expression; Suppression of antioxidant enzyme activity (SOD and CAT); Increased superoxide anion generation; ER stress → p-PERK–p-IRE1α–ATF4–CHOP; Induction of apoptosis via increased BAX and cleaved caspase-3 expression and decreased BCL-2 levels	Activation of endoplasmic reticulum stress-mediated signaling pathway; Overcoming of oxaliplatin resistance through increased apoptosis in resistant cancer cells	[100]
Colorectal cancer HT-29 cells	5-Fluorouracil (1 mM)	1 mM	PI3K/AKT/NF-κB → suppression of PI3K/AKT and NF-κB/iNOS signaling; Melatonin may act through modulation of survival pathways	Increased apoptotic cell population, caspase-3 and caspase-9 activation, and cytochrome c release; Reduction of cell viability and proliferation; Synergistic enhancement of melatonin combined with 5-Fluorouracil-induced chemotherapy; ↑ ROS generation induced by combined treatment	[101]

Note: Abbreviations used: 4E-BP1—eukaryotic translation initiation factor 4E-binding protein 1; ATF4—activating transcription factor 4; AP-2β—activator protein 2 beta; BAX—B-cell lymphoma 2-associated X protein; BiP—binding immunoglobulin protein; Ca^2+^—calcium ions; CAT—catalase; CHOP—C/EBP homologous protein; COX-2—cyclooxygenase 2; ERK—extracellular signal-regulated kinase; GSK3β—glycogen synthase kinase 3 beta; hTERT—human telomerase reverse transcriptase; iNOS—inducible nitric oxide synthase;LC3-II—microtubule-associated protein 1 light chain 3-II; MT2—melatonin receptor type 2; NF-κB—nuclear factor kappa-light-chain-enhancer of activated B cells; p-IRE1α—phosphorylated inositol-requiring enzyme 1 α; p62—sequestosome 1; p70S6K—p70 S6 kinase; PDGFR-β—platelet-derived growth factor receptor beta; PDI—protein disulfide isomerase; PrPC—cellular prion protein; Sub-G1—sub-G1 phase of the cell cycle; SMAC—second mitochondria-derived activator of caspases; SOD—superoxide dismutase; STAT3—signal transducer and activator of transcription 3; TFAM—mitochondrial transcription factor A; TFB1M—mitochondrial transcription factor B1; TFB2M—mitochondrial transcription factor B2.

#### 3.4.4 Integration of Chronotherapy and Combination Strategies

In the context of chronobiology, the concept of chronotherapy is gaining increasing importance, assuming the adjustment of the timing of anticancer drug administration to the body’s circadian rhythm. Proper synchronization of therapy with physiological fluctuations in clock gene expression may enhance the efficacy of drugs and reduce their toxicity in patients [90]. Current research directions include the design of multifunctional ligands that, in addition to activating MT1/MT2 receptors, also act on other targets such as serotonin receptors or enzymes of the endocannabinoid system, which may enhance their therapeutic efficacy [87].

Melatonin represents a promising component of adjuvant therapy in oncology, combining antiproliferative, proapoptotic, antioxidant, and circadian rhythm–modulating effects. Its pharmacological properties, including a short half-life and extensive hepatic metabolism, justify the need for using different pharmaceutical formulations, such as immediate-release and prolonged-release preparations, to achieve optimal clinical outcomes. Increasing evidence indicates that melatonin not only supports circadian rhythm regulation but also modulates the expression of clock genes and numerous signaling pathways associated with carcinogenesis, making it an important factor in cancer chronotherapy strategies. Due to the pleiotropic mechanism of melatonin action, involving the modulation of multiple signaling pathways related to proliferation, apoptosis, OS, and cellular metabolism, the appropriate selection of dose and administration regimen should be tailored to the tumor type, therapeutic goal, and type of combination therapy used.

## 4 Experimental and Clinical Evidence in Integrative Oncology

### 4.1 Preclinical Evidence

Preclinical studies demonstrate that modulation of ROS significantly impacts cancer progression by regulating apoptosis, altering tumor metabolism, and shaping antioxidant adaptation in cancer cells. Evidence from *in vitro* experiments and animal models shows that increased ROS production can activate cell death pathways and promote genomic instability, thereby inhibiting tumor growth in early stages [102,103]. However, prolonged exposure to elevated ROS levels induces the expression of antioxidant enzymes, favoring the selection of resistant clones and potentially promoting tumor progression [104].

In recent years, significant progress has been made in understanding the therapeutic potential of melatonin as an OS modulator in oncology. Recent preclinical studies indicate that melatonin not only reduces excessive ROS levels by directly scavenging free radicals but also modulates the expression of antioxidant enzymes [105–107]. In models of breast and colon cancer, melatonin has been shown to stabilize mitochondrial respiratory chain function, reduce lipid peroxidation, and restore redox homeostasis in healthy cells [108,109]. Simultaneously, it selectively increases OS in cancer cells. These mechanisms include regulation of apoptotic proteins, inhibition of the AKT and NF-κB signaling pathways, and potential induction of ferroptosis, according to recent studies [60,110].

Animal studies further support the therapeutic importance of redox modulation [111]. Reducing OS, for example, by overexpressing mitochondrial CAT, significantly reduced metastatic potential. Metabolic interventions, such as inhibition of oxidative phosphorylation, led to profound remodeling of tumor metabolism, alleviating hypoxia and improving response to therapy [112]. High levels of ROS can also induce autophagy and various forms of regulated cell death, thus offering additional therapeutic opportunities [113]. Selected preclinical studies on melatonin and its modulation of OS in cancer models are summarized in Table 4.

**Table 4:** Summary of key preclinical studies investigating the effects of melatonin and other ROS-modulating strategies in cancer models.

Research Model	Study Aim	Key Findings	ROS/OS Mechanism	Ref.
rats, MTX–induced neurotoxicity	assessment of the effect of melatonin on the decrease in antioxidant activity induced by methotrexate	melatonin increases the activity of endogenous antioxidant enzymes, reduces OS, and improves neurogenesis	↓ ROS, ↑ SOD, CAT, GSH; protection of healthy tissues	[105]
birds (ovarian model), dexamethasone damage	investigation into whether melatonin alleviates oxidative damage to the gonads	limiting lipid peroxidation, restoring ovarian cell function	↓ MDA, ↑ FOXO1–dependent antioxidant regulation	[106]
rats, “chemo brain” after doxorubicin	assessment of the neuroprotective effect of melatonin on DOX-induced OS	melatonin protects the brain, kidneys, and liver from OS and normalizes behavioral parameters	activation of the Nrf2/p53–SIRT1 axis, ↓ ROS	[107]
rats, DMBA-induced breast cancer	assessment of the effect of melatonin + Zn on lipid peroxidation in tissues	Zn + melatonin reduce MDA and improve redox status in various tissues	↓ lipid peroxidation, mitochondrial stabilization	[108]
colon cancer cells, healthy cells (gingiva, MSC)	selectivity evaluation of melatonin cytotoxicity	melatonin has a cytotoxic effect on cancer cells while protecting healthy cells	selective ↑ ROS in cancer cells; ↓ ROS in healthy cells	[109]
gastric cancer cells (AGS)	elucidation of the melatonin-induced apoptosis pathway	melatonin activates PERK/eIF2 α and inhibits HSF1/NF-κB → strong induction of apoptosis	↑ ROS, NF-κB inhibition; ER stress pathway activation	[110]
cancer cells—multiple forms of death	evaluation of the role of ROS-induced lipid peroxidation	ROS determine the switch between apoptosis, autophagy and ferroptosis	ROS-dependent lipid peroxidation; ferroptosis induction	[113]

Note: Abbreviations used: AGS—gastric adenocarcinoma cells; DOX—doxorubicin; FOXO1—forkhead box protein O1; HSF1—heat shock transcription factor 1; MDA—malondialdehyde; MSC—mesenchymal stem cells; MTX—methotrexate; NF-κB—nuclear factor kappa-light-chain-enhancer of activated B cells; p53—tumor protein p53; SIRT1—silent information regulator 1.

Taken together, these findings highlight that targeted manipulation of the ROS-antioxidant balance represents a promising strategy in both cellular and animal models, and melatonin emerges as a desirable candidate for redox-based anticancer interventions.

### 4.2 Clinical Applications

Modulating redox balance is increasingly recognized as an adjunctive role in integrative oncology, complementing chemotherapy, radiotherapy, and immunotherapy. Clinical data indicate that targeting redox balance can enhance the cytotoxic effects of conventional treatments, promote immunogenic cell death, and modulate immune checkpoint expression, thereby increasing the efficacy of immunotherapy [114–116]. Interventions aimed at reducing OS, including supplementation with antioxidants such as vitamins C and E, flavonoids, and alpha-lipoic acid, have been shown to reduce treatment-related toxicity and improve patients’ quality of life [115,117,118].

In recent years, accumulating clinical evidence has strengthened the rationale for incorporating melatonin into integrated cancer care. Studies have shown that melatonin can improve tolerance to cancer therapy, alleviate fatigue, normalize circadian rhythms, and support sleep quality in patients [119,120]. Observational analyses have also demonstrated that melatonin use was associated with a reduced incidence of chemotherapy-induced neuropathy and myelosuppression [121]. Importantly, studies confirm that melatonin administration in patients undergoing radiotherapy or chemotherapy is associated with improved quality of life and fewer adverse events [122–125]. However, additional controlled studies are needed to determine its effect on endpoints such as overall survival.

Notably, it should be emphasized that the majority of available clinical studies evaluating melatonin in oncology rely predominantly on surrogate endpoints, such as quality of life, cancer-related fatigue, sleep parameters, or selected OS biomarkers. While these outcomes are clinically relevant from a supportive care perspective, they do not directly translate into hard oncologic endpoints, including progression-free survival or overall survival. Consequently, the strength of clinical inferences regarding melatonin’s disease-modifying or oncostatic effects remains limited.

Preliminary reports also suggest a potential collaboration between melatonin and immunotherapy, particularly through modulation of the tumor microenvironment, reduction of interleukin-6 (IL-6) expression, and suppression of myeloid-derived suppressor cell (MDSC) activity [126]. However, these results remain exploratory and require further validation. Selected clinical studies indicate that ROS-modulating strategies can prolong overall survival and progression-free survival, especially when combined with immunotherapeutic approaches. Simultaneously, integrative methods such as rehabilitation and phytotherapy contribute to improved functional status, psychological well-being, and treatment tolerance [115,127]. The most essential available clinical studies evaluating melatonin as adjunctive therapy in cancer patients are summarized in Table 5. However, these trials should be interpreted with caution, as most were not powered to detect differences in long-term oncologic outcomes.

**Table 5:** Contemporary randomized controlled clinical trials evaluating melatonin as adjunctive therapy in oncology.

Population	Melatonin Dose	Study Objective	Key Clinical Findings	Redox/Adjunctive Therapy Conclusions	Ref.
breast cancer patients undergoing RT	20 mg/d	reduced fatigue and improved QoL	no differences vs. placebo; good tolerance	negative results at high doses highlight the need to refine dosing regimens	[123]
FOLFOX (colon cancer)	20 mg/d	prevention of oxaliplatin-induced CIPN	significantly less grade 3 neuropathy vs. placebo	melatonin protects healthy tissues (neurons) without weakening the effect of chemotherapy	[121]
breast cancer during chemotherapy	1 mg/d	CRF reduction	moderate improvement in fatigue, no side effects	effectiveness may depend on the dose—a low dose has a symptomatic effect	[120]
women with breast cancer undergoing adjuvant chemotherapy and radiotherapy	18 mg/d	evaluation of the effect of melatonin on CRF associated with breast cancer and adjuvant treatment	significantly greater reduction in fatigue severity in the melatonin group vs. placebo after treatment completion; good tolerability without severe melatonin-related adverse events	long-term supplementation at a moderately high dose may alleviate CRF without impairing the efficacy of anticancer treatment, probably by modulating the neuro-immuno-endocrine axis and OS	[128]
breast cancer patients undergoing chemotherapy	20 mg/d	assessment of cognitive function, sleep quality, mood, and treatment tolerance during chemotherapy	improvement in sleep quality and reduction of insomnia; reduced depressive symptoms; trends toward improved cognitive function; excellent tolerability	melatonin supports neurocognitive and psychological stability during chemotherapy, likely through circadian regulation and antioxidant/neuroprotective mechanisms	[129]

Note: Abbreviations used: CIPN—chemotherapy-induced peripheral neuropathy; CRF—cancer-related fatigue; FOLFOX—folinic acid, fluorouracil and oxaliplatin; mg/d—milligrams per day; QoL—quality of life; RCT—randomized controlled trial; RT—radiotherapy.

The observations described above highlight the therapeutic potential of redox-modulating interventions, particularly melatonin, as adjunctive strategies in integrative oncology. These interventions have the potential to enhance treatment efficacy, reduce toxicity, and improve overall patient well-being.

It should also be emphasized that the available clinical studies employ highly heterogeneous melatonin doses, formulations, and treatment schedules, ranging from low physiological supplementation to pharmacological dosing, with substantial variability in timing, duration, and formulation (immediate- vs. prolonged-release). This heterogeneity precludes the establishment of a consistent dose–response relationship and significantly limits direct comparisons across studies. Consequently, the translational interpretation of clinical findings remains constrained, and current evidence does not allow for the identification of an optimal therapeutic dose or dosing regimen in oncologic settings.

Despite these encouraging clinical observations, an important unresolved issue remains the pronounced heterogeneity of melatonin dosing strategies across available studies. Clinical trials differ substantially with respect to administered doses, formulation (immediate-release vs. prolonged-release), timing of administration, and duration of treatment, reflecting divergent therapeutic goals ranging from circadian regulation and symptom control to potential oncostatic effects [130]. Importantly, a consistent dose–response relationship has not been established in oncological settings [131]. Lower doses have been primarily associated with improvements in sleep quality and cancer-related fatigue, whereas higher pharmacological doses have been explored in the context of redox modulation and treatment tolerance, often with variable or neutral effects on hard oncologic endpoints [131]. This variability complicates cross-study comparisons and limits the formulation of evidence-based dosing recommendations, highlighting the need for future trials specifically designed to address dose optimization, pharmacokinetics, and patient stratification.

### 4.3 Administration Methods and Pharmaceutics of Melatonin

In oncological settings, melatonin is administered predominantly via the oral route, most commonly in immediate-release or prolonged-/controlled-release formulations [132]. Immediate-release preparations are typically used to target circadian regulation and sleep-related outcomes, whereas prolonged-release formulations are designed to provide more stable nocturnal plasma concentrations and may be more relevant for sustained biological effects, including redox modulation [133]. Oral bioavailability of melatonin is known to be variable and influenced by formulation, dosing, and timing of administration, which may partly contribute to the heterogeneity of clinical outcomes observed across studies [134]. Recent clinical and translational reports emphasize that pharmacokinetic considerations, including formulation type and administration timing, are critical for interpreting therapeutic responses in cancer patients [132,133,135]. However, despite growing interest in adjunctive melatonin use in oncology, standardized pharmaceutic strategies and administration protocols remain insufficiently defined, underscoring the need for well-designed studies integrating pharmacokinetics with clinically meaningful endpoints.

### 4.4 Integrative Oncology Context—Limitations and Controversies

Despite the growing number of studies on melatonin in oncology, several important limitations continue to hinder a clear interpretation of the available evidence. This section focuses on limitations inherent to the current evidence base, which directly inform, but should be conceptually distinguished from, the organizational and policy-related considerations discussed in Section 5. A major challenge is the substantial heterogeneity across clinical trials, including wide variation in melatonin doses (from 5 mg to over 50 mg daily) and the frequent absence of standardized formulations. Differences in timing and duration of administration further complicate comparisons between studies. Many investigations are based on small patient cohorts, limiting statistical power, while the lack of standardized biomarkers for assessing redox modulation or clinical benefit makes it difficult to determine which patients are most likely to respond [120,122,136].

Moreover, many clinical investigations prioritize patient-reported outcomes and biochemical markers of OS rather than survival-based endpoints, which further limits the extrapolation of these findings to definitive oncologic benefit.

In addition, the lack of standardized dosing strategies and the absence of dose–response analyses further complicate the interpretation of clinical outcomes and weaken the translational relevance of existing evidence.

Thus, the inclusion of melatonin as an adjunctive therapy requires refined dosing recommendations and careful evaluation of potential interactions with anticancer treatments. It will also be necessary to identify patient subgroups most likely to benefit. These challenges are amplified by an ongoing debate about the safety of antioxidant use during ROS-generating therapies such as chemotherapy and radiotherapy—a common concern in clinical oncology. Nevertheless, accumulating evidence suggests that melatonin functions not merely as a classical antioxidant but as a selective regulator of OS, attenuating damage to healthy tissues while preserving, or even enhancing, the cytotoxic effects of anticancer treatments [107,121,137].

Melatonin’s potential advantages include a favorable safety profile, minimal risk of drug interactions, and a broad spectrum of biological actions encompassing antioxidant, anti-inflammatory, immunomodulatory, and direct anticancer effects. However, these strengths are counterbalanced by persistent limitations in the evidence base. Clinical studies remain inconsistent, with a paucity of large randomized trials, methodological heterogeneity, and considerable variability in dosage, formulation, and outcome measures. As a result, despite encouraging preclinical findings and partial clinical benefits, melatonin has not yet been widely implemented in routine oncology practice.

Future directions should include the development of personalized treatment strategies and the integration of melatonin with other evidence-based supportive interventions. In summary, melatonin is a promising, well-tolerated and mechanistically versatile adjuvant; however, its full clinical implementation will require more rigorously designed studies, standardized procedures and consistent clinical guidelines.

## 5 Strategic Management Perspectives

### 5.1 Strategic Implementation: Institutional Decision-Making, Barriers and Facilitators to Adoption

In recent years, the incorporation of melatonin as an adjunctive modality within integrative oncology has attracted growing scientific and clinical interest due to its potential to enhance therapeutic efficacy and mitigate adverse effects associated with standard cancer treatments. Although traditionally recognized for its role in circadian rhythm regulation, melatonin also exhibits potent antioxidant activity and influences a broad spectrum of biological pathways that underpin tumorigenesis, disease progression, and treatment responsiveness [138]. While the evidentiary limitations of melatonin use in oncology are discussed in detail in Section 4, the present section focuses on how these constraints translate into practical barriers and facilitators at the institutional and organizational level.

A growing body of preclinical and early clinical evidence suggests that melatonin may enhance the effects of chemotherapeutic agents while simultaneously attenuating treatment-related toxicities, which has direct implications for institutional decision-making, supportive care planning, and integrative oncology program development. Mechanistically, melatonin has been shown to reduce tumor growth and suppress cancer stemness through the modulation of key intracellular signaling networks, including ERK, p38 MAPK, and β-catenin pathways [139]. Moreover, studies conducted in colorectal, gastric, lung, and breast malignancies demonstrate that the addition of melatonin to standard oncologic regimens is associated with improvements in tumor control, treatment tolerance, and survival outcomes [66,140,141].

Despite these promising findings, the institutional implementation of melatonin remains limited. One major constraint involves the absence of standardized dosing recommendations and consensus-based clinical guidelines, which contributes to uncertainty among clinicians regarding melatonin’s therapeutic indications, safety profile, and optimal use scenarios. Although the literature consistently supports a favorable safety profile, with positive outcomes observed across a range of dosages, clear clinical frameworks delineating appropriate patient selection and dosing strategies are lacking [142–145].

Several facilitators may enable broader integration of melatonin into oncology practice. Clinical and patient advocacy, supported by randomized controlled trials and systematic reviews, has increasingly highlighted melatonin’s capacity to modulate inflammatory processes, enhance quality of life, and complement established antineoplastic modalities. Notably, these facilitators are grounded primarily in clinical findings related to supportive care outcomes, including reduced treatment-related toxicity, improved sleep quality, and attenuation of cancer-related fatigue, rather than consistent effects on survival-based endpoints. Articulating melatonin’s mechanistic compatibility with existing therapeutic approaches may further support its adoption as part of comprehensive integrative oncology programs [144,146].

Patient education also represents an essential dimension of implementation efforts. Improved patient awareness regarding the potential benefits, safety, and rationale for melatonin use may increase acceptance and encourage clinician engagement. Parallel efforts emphasizing interdisciplinary collaboration and translational research can facilitate the development of evidence-based protocols, enabling the translation of mechanistic and early-phase clinical findings into real-world oncology settings [120,147].

Regulatory discrepancies between countries and the lack of standardized formulations further complicate implementation. Therefore, while melatonin appears promising as a safe and mechanistically sound adjunctive therapy, its routine adoption should be approached cautiously and guided by ongoing research and evolving evidence-based frameworks [122,148].

Although melatonin demonstrates considerable potential as an adjunctive agent capable of improving clinical outcomes and reducing treatment-related toxicities, its integration into mainstream oncology remains hampered by institutional, perceptual, and regulatory barriers. Targeted strategies including the dissemination of clinical guidelines, refinement of dosing frameworks, and continued investigation into mechanistic and clinical efficacy will be essential to support informed clinical adoption and to optimize the therapeutic and organizational value of melatonin within integrative oncology [149,150].

These organizational considerations are directly informed by the experimental and clinical evidence summarized in Section 4, particularly studies demonstrating improvements in treatment tolerance, quality of life, and selected OS markers following melatonin supplementation.

### 5.2 Organizational Strategies: Interdisciplinary Teams, Training and Long-Term Planning

The integration of melatonin into cancer care as an adjunctive therapeutic modality requires well-designed organizational models that support interdisciplinary teamwork, targeted professional training, and long-term strategic planning, and is directly informed by the clinical and translational evidence summarized in Section 4, particularly studies demonstrating improvements in treatment tolerance, patient-reported outcomes, and quality of life rather than consistent effects on survival-based endpoints.

The integration of melatonin into cancer care as an adjunctive therapeutic modality requires well-designed organizational models that support interdisciplinary teamwork, targeted professional training, and long-term strategic planning. Given its potential to improve treatment outcomes and enhance quality of life, as demonstrated by multiple clinical studies and systematic reviews, the successful incorporation of melatonin into routine oncology practice necessitates a structured approach that reflects the complexity of contemporary cancer care delivery [148].

Interdisciplinary collaboration represents a central pillar of effective implementation. Coordinated involvement of oncologists, palliative care specialists, pharmacists, and nursing personnel enables the development of holistic treatment plans tailored to individual patient needs. Such models must align with the financial and operational realities of oncology services, ensuring that melatonin supplementation is perceived as providing added clinical value rather than imposing additional logistical burden [151]. Evidence suggests that strengthening communication and cooperation among healthcare professionals contributes to improvements in patient outcomes and care satisfaction [152]. Incorporating patient perspectives into decision-making further enhances treatment acceptance and adherence. Shared decision-making around adjunctive therapies, including melatonin, has been recognized as integral to supporting patient engagement and ensuring that care is responsive to evolving individual preferences. The establishment of multidisciplinary task groups involving healthcare providers and community representatives may facilitate the development of implementation strategies that remain adaptable to patient needs over time [153–155].

Training and education constitute additional prerequisites for successful organizational adoption. Clinicians must possess sufficient knowledge of melatonin’s mechanisms of action, dosing strategies, and safety profile to appropriately contextualize its use within multimodal cancer treatment. Targeted educational initiatives can help address knowledge gaps and foster confidence among healthcare professionals in implementing melatonin as an adjunctive agent [156]. Recommendations to enhance palliative care competencies within oncology training curricula further underscore the importance of preparing practitioners to integrate complementary approaches, such as melatonin, earlier in the care continuum. Ongoing professional development programs are essential to maintaining awareness of current research and supporting evidence-informed clinical decision-making [157].

Long-term planning is likewise essential to ensure sustainable incorporation of melatonin into oncology services. The development of clear clinical guidance on indications, timing, and dosing can facilitate clinician uptake and contribute to standardization of practice. Evidence-based clinical pathways informed by available studies demonstrating the therapeutic benefits of melatonin may support its broader adoption within integrated care models [158]. Organizational planning should additionally address resource allocation and monitoring frameworks to assess effectiveness, adherence, and patient-reported outcomes over time. Institutional commitment and leadership engagement are critical to sustaining integration efforts and ensuring that protocols can be adapted as new evidence emerges [159,160].

The integration of melatonin into cancer care requires a multi-level organizational strategy encompassing interdisciplinary collaboration, systematic training, and proactive long-term planning. By fostering coordinated teamwork, supporting clinician education, and establishing structured implementation frameworks, oncology services may more effectively harness the therapeutic potential of melatonin as an adjunctive intervention to enhance patient outcomes within integrative oncology [161,162].

### 5.3 Policy and System-Level Perspectives: Integrating Evidence-Based Adjunctive Therapies

The incorporation of evidence-based adjunctive therapies, such as melatonin, into oncology practice requires the development of robust policy- and system-level frameworks that support integration across diverse care settings. These frameworks should promote coordinated stakeholder engagement, expand opportunities for education and training, and address the structural barriers that hinder widespread implementation [163]. Given the growing recognition of melatonin’s therapeutic potential, such strategies are essential to ensure that its use is embedded within comprehensive cancer care rather than remaining peripheral to standard treatment pathways. This recognition is largely based on translational and early clinical evidence summarized in Section 4, which supports melatonin’s role as a safe adjunctive intervention within supportive and integrative oncology frameworks. The development of policies and structured guidelines for incorporating supportive and integrative therapies into oncology care has been emphasized in contemporary clinical frameworks [164].

Policy structures designed to facilitate integration must acknowledge supportive care as an essential component of cancer management. Supportive care, encompassing symptom control, psychosocial support, and strategies aimed at optimizing quality of life, has been shown to meaningfully improve treatment tolerance and patient-reported outcomes [165]. Recent frameworks emphasize that supportive care should function as a core element of cancer service planning and delivery, rather than as an optional adjunct. This conceptual shift provides an opportunity to systematize the role of melatonin alongside other supportive care interventions and to embed it within established clinical pathways, ultimately enabling more standardized and equitable access to adjunctive therapeutics [166].

Interdisciplinary collaboration is similarly critical to the successful integration of melatonin into oncology care. Networks involving oncologists, palliative care specialists, nutritionists, pharmacists, and mental health professionals can enhance care coordination, streamline communication, and ensure treatment plans reflect the multifaceted needs of patients with cancer [167]. Policy mechanisms supporting cross-sectoral collaboration, including cooperation between public and private providers, may broaden service availability and improve continuity of care. Such structures are essential to support melatonin integration within existing clinical infrastructures and to promote consistency in its therapeutic use [168].

Training and capacity-building initiatives are also indispensable. Enhancing healthcare professionals’ understanding of melatonin’s mechanisms of action, safety profile, and potential role within multimodal cancer therapy can improve clinician confidence and facilitate evidence-informed prescribing practices [169]. Educational strategies may include targeted professional development, incorporation of integrative oncology content into medical curricula, and university-industry partnerships that support research translation into practice. Such initiatives can collectively strengthen the clinical workforce’s readiness to utilize melatonin as part of supportive oncology [170,171].

Long-term planning and continuous evaluation are necessary to sustain and refine integration efforts. Health systems should develop mechanisms to monitor effectiveness, adherence, and patient-reported outcomes, including formal registries and standardized assessment protocols [172,173]. These data can, in turn, guide iterative protocol development, enabling dynamic adaptation as new evidence emerges. Dedicated funding streams to support research examining adjunctive therapies, including melatonin, will be essential for improving clinical guidance and narrowing current evidence gaps [174,175].

**Figure 3:**
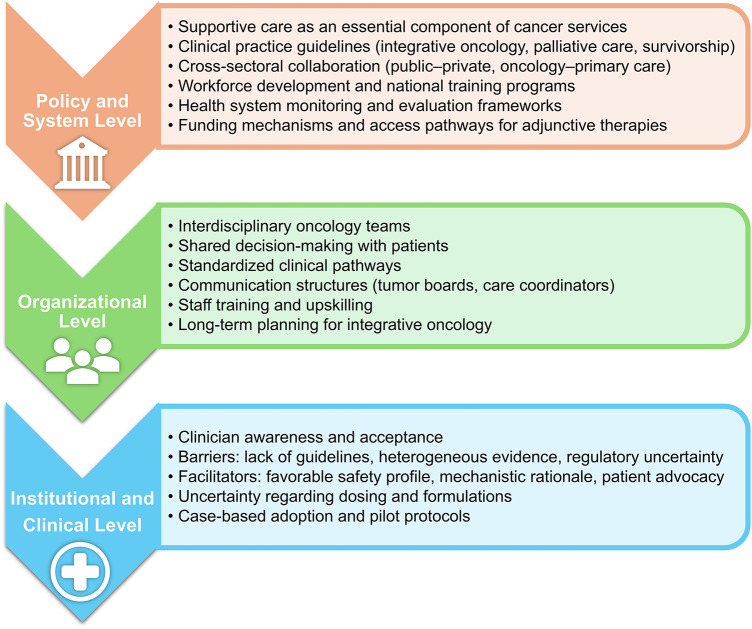
Multilevel framework for the implementation of melatonin as an adjunctive therapy in integrative oncology. The diagram summarizes three interconnected levels that influence the adoption of melatonin in cancer care: the policy and system level, encompassing supportive care policies, clinical practice guidelines, cross-sectoral collaboration, workforce development, and funding or access mechanisms; the organizational level, including interdisciplinary oncology teams, standardized clinical pathways, communication structures, shared decision-making with patients, and staff training; and the institutional/clinical level, involving clinician awareness and acceptance, evidence gaps, regulatory uncertainty, facilitators such as melatonin’s favorable safety profile and mechanistic rationale, as well as uncertainty regarding dosing and formulation and case-based early adoption strategies. Together, these levels illustrate the strategic, operational, and systemic factors shaping the evidence-based integration of melatonin into oncology practice. The figure was created using Microsoft PowerPoint 2021.

The successful incorporation of melatonin into cancer care is contingent upon policy-level responses that mitigate systemic barriers, including limited resources, lack of standardized treatment frameworks, and fragmented care delivery. Policies promoting integrated service models across primary, secondary, and tertiary care may help ensure equitable access to adjunctive therapies for all patients, regardless of treatment setting. A coherent, multilevel policy agenda that enables collaboration, supports clinician training, and embeds structured evaluation processes is therefore critical to realizing the full therapeutic value of melatonin within integrative oncology and to improving patient outcomes [164,176–178]. A visual synthesis of the institutional, organizational, and system-level determinants of melatonin implementation is presented in Fig. 3.

## 6 Future Directions and Research Gaps

Although substantial progress has been made in understanding melatonin’s biological actions in cancer, important gaps remain across molecular, clinical and organizational dimensions. At the mechanistic level, further research is required to clarify the context-dependent redox effects of melatonin, including determinants of its selective pro-oxidant activity in cancer cells vs. antioxidant protection in healthy tissues. More work is also needed to delineate mitochondrial targets, chronobiological regulation and interactions with key oncogenic pathways beyond those already characterized. Priority experimental approaches include comparative *in vitro* and *in vivo* models integrating redox profiling, mitochondrial functional assays and circadian phase manipulation, with particular emphasis on signaling nodes such as PI3K/AKT/mTOR, Nrf2–Keap1 and p53-dependent stress responses.

Clinically, many limitations previously discussed—including heterogeneous dosing regimens, differences between formulations, small sample sizes and inconsistent outcome measures—highlight the need for adequately powered randomized trials with standardized protocols. Future studies should prioritize dose-finding research, pharmacokinetic and pharmacodynamic profiling and controlled evaluations of melatonin in combination with immunotherapies, targeted agents and radiotherapy. From a study design perspective, adaptive phase II trials and randomized controlled studies incorporating predefined dosing schedules and biomarker-stratified patient cohorts would be particularly informative, especially in tumor types where OS modulation and circadian disruption are clinically relevant, such as lung, breast, gastrointestinal and selected hematological malignancies. Biomarker-driven approaches, including redox signatures, mitochondrial phenotypes and circadian parameters, may help identify patient subgroups most likely to benefit. Candidate biomarkers may include oxidative DNA damage markers (e.g., 8-oxo-dG), antioxidant enzyme activity profiles, mitochondrial membrane potential, clock gene expression patterns and treatment-induced changes in circadian rhythm stability.

From an implementation perspective, a major research gap concerns the translation of biological and clinical knowledge into routine oncology practice. Future work should examine organizational readiness, clinician awareness, communication strategies in shared decision-making and the development of clear clinical pathways for adjunctive melatonin use. Mixed-methods research combining qualitative implementation studies with prospective observational cohorts may be particularly useful to assess feasibility, adherence, and real-world effectiveness across different oncology care settings. System-level considerations—including regulatory guidance, quality standards for over-the-counter formulations and health-economic evaluations—will also be essential to support responsible and evidence-based integration. Addressing these multilevel gaps through coordinated, interdisciplinary research will be critical for realizing the full therapeutic and clinical potential of melatonin in integrative oncology.

## 7 Conclusions

Melatonin has emerged as a biologically versatile and clinically relevant molecule with significant potential in integrative oncology. Its actions span multiple levels of cancer biology, including redox regulation, mitochondrial stabilization, modulation of cell-cycle checkpoints, interference with oncogenic signaling, and support of immunological homeostasis. Evidence from preclinical studies consistently demonstrates that melatonin can enhance the anticancer effects of chemotherapy, radiotherapy, targeted agents, and immunotherapies while simultaneously protecting healthy tissues from treatment-induced oxidative damage. Early clinical findings further suggest improvements in treatment tolerance, symptom burden, sleep regulation, and selected oncologic outcomes, although methodological heterogeneity still limits the strength of conclusions.

Importantly, the therapeutic relevance of melatonin extends beyond molecular and clinical mechanisms. Organizational factors, clinician awareness, regulatory clarity, and system-level strategies play a crucial role in determining whether evidence-based interventions can be successfully adopted into oncology practice. Integrating melatonin into routine care, therefore, requires a structured, multilevel approach that includes standardized protocols, interdisciplinary engagement, patient-centered communication, and alignment with supportive-care frameworks.

Overall, melatonin represents a promising adjunctive option for enhancing therapeutic efficacy, reducing toxicity, and improving patient well-being within integrative oncology. Future progress will depend on high-quality randomized trials, optimized dosing frameworks, harmonized outcome measures, and implementation research that bridges biomedical evidence with organizational and health-system realities. A coordinated effort across molecular science, clinical practice, and healthcare management will be essential to translate melatonin’s therapeutic potential into consistent and accessible oncology care.

## Data Availability

Not applicable.
